# Effectiveness of a posture education program in high school students: A randomized controlled trial protocol

**DOI:** 10.1016/j.mex.2026.104006

**Published:** 2026-06-13

**Authors:** Jeyanthi Valaitham, Asfarina Zanudin, Nurfaradilla Mohamad Nasri, Ayiesah Ramli

**Affiliations:** a Physiotherapy Programme, Centre for Rehabilitation and Special Needs Studies, Faculty of Health Sciences, Universiti Kebangsaan Malaysia, Jalan Raja Muda Abdul Aziz, 50300, Kuala Lumpur, Wilayah Persekutuan Kuala Lumpur, Malaysia; b Centre of Educational Leadership and Policy, Faculty of Education, Universiti Kebangsaan Malaysia, Jalan Raja Muda Abdul Aziz, 50300, Kuala Lumpur, Wilayah Persekutuan Kuala Lumpur, Malaysia

**Keywords:** Adolescents, Posture education, Randomized controlled trial, School-based intervention, Postural alignment, Cardiorespiratory fitness, Ergonomics

## Abstract

Adolescents are increasingly exposed to sedentary behaviours and poor ergonomic environments that may adversely affect posture, aerobic capacity, and psychological well-being. This article describes a standardized protocol for implementing and evaluating an 8-week Posture Education Program (PEP) among high school students aged 14–17 years using a randomized controlled trial design. The intervention includes postural awareness, core strengthening, ergonomic correction, guided movement, and reflective behavioural activities delivered during physical education sessions. The control group will continue standard physical education without posture-specific training. The program was developed using the ADDIE instructional framework, while the International Classification of Functioning, Disability and Health framework guided outcome selection. Primary outcomes include postural alignment, aerobic capacity, and lung function. Secondary outcomes include physical activity, psychological well-being, and ergonomic knowledge and behaviour. This protocol provides a reproducible framework for school-based posture education interventions and integrates multidimensional assessment of postural, cardiorespiratory, behavioural, and psychological outcomes within a school-based adolescent population.

Structured 8-week school-based posture education intervention integrated into physical education sessions.

Multidimensional assessment of posture, aerobic capacity, lung function, and psychological well-being.

Reproducible randomized controlled trial protocol guided by ADDIE and ICF frameworks.

## Graphical abstract

**Figure:**
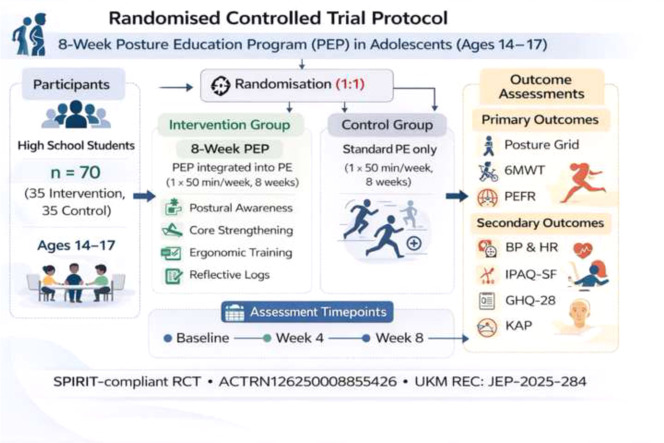

Specifications table

**Table:** 

**Subject area**	Medicine and Dentistry
**More specific subject area**	School-based physiotherapy / adolescent posture intervention
**Name of your method**	Posture Education Program (PEP) for High School Students
**Name and reference of original method**	Developed using the ADDIE instructional design model and informed by ergonomic and posture education literature.
**Resource availability**	Peak flow meter, automatic blood pressure monitor, posture grid, IPAQ-SF, GHQ-28, KAP questionnaire, stopwatch, standardized 6-minute walk test (6MWT) track setup, and intervention session materials.

## Background

Adolescents are [1] increasingly exposed to lifestyle factors that contribute to suboptimal postural alignment, including prolonged screen use, sedentary behaviour, and inadequate ergonomic awareness in school and home environments [2,3]. During secondary school years, students often engage in prolonged static sitting and device-based learning, which may influence musculoskeletal development and postural habits. Emerging evidence suggests that postural deviations in adolescents may be associated not only with musculoskeletal discomfort but also with altered respiratory function and reduced pulmonary efficiency [1,4]. In addition, reduced physical activity and increased sedentary behaviour during adolescence have been linked to cardiovascular risk profiles and psychosocial stress indicators [3,5]. These findings highlight the potential multidimensional health implications of sustained postural imbalance during formative developmental years.

Several corrective exercises and posture-focused interventions have been implemented in adolescent populations. For example, corrective exercise programs focusing on thoracic kyphosis and upper-quadrant alignment have demonstrated improvements in postural parameters and body image among adolescents with hyperkyphosis [6]. Nevertheless, structured, school-based posture education programs designed for preventive use in generally healthy adolescents remain limited. Furthermore, few protocols have integrated postural education with objective measures of cardiorespiratory health and standardized psychological well-being assessments within a single framework. Existing school-based interventions have commonly focused on general physical activity, fitness promotion, corrective exercise, or ergonomic awareness as separate components. However, few published protocols have combined posture education, progressive postural exercise, ergonomic behaviour training, and psychological well-being assessment within a single school-based randomized controlled trial. In addition, many posture-related interventions focus on adolescents with existing postural deviations, whereas preventive programs for generally healthy high school students remain less clearly described. The present protocol addresses this gap by providing a structured, theory-informed, and reproducible intervention that can be integrated into scheduled physical education sessions without major disruption to the school timetable. Several posture-focused and corrective exercise interventions have been conducted among adolescent populations; however, their intervention approaches, outcome domains, and implementation contexts vary considerably. For example, Mokhtaran et al [6] evaluated two corrective exercise approaches among adolescent schoolgirls with hyperkyphosis over an 8-week intervention period using upper-quadrant posture and body image as primary outcome measures. The study reported improvements in postural alignment and body image following the intervention. However, the study focused primarily on adolescents with existing postural deviations and did not assess cardiorespiratory outcomes, ergonomic behaviour, or psychological well-being within a preventive school-based framework.

**Fig. 1:**
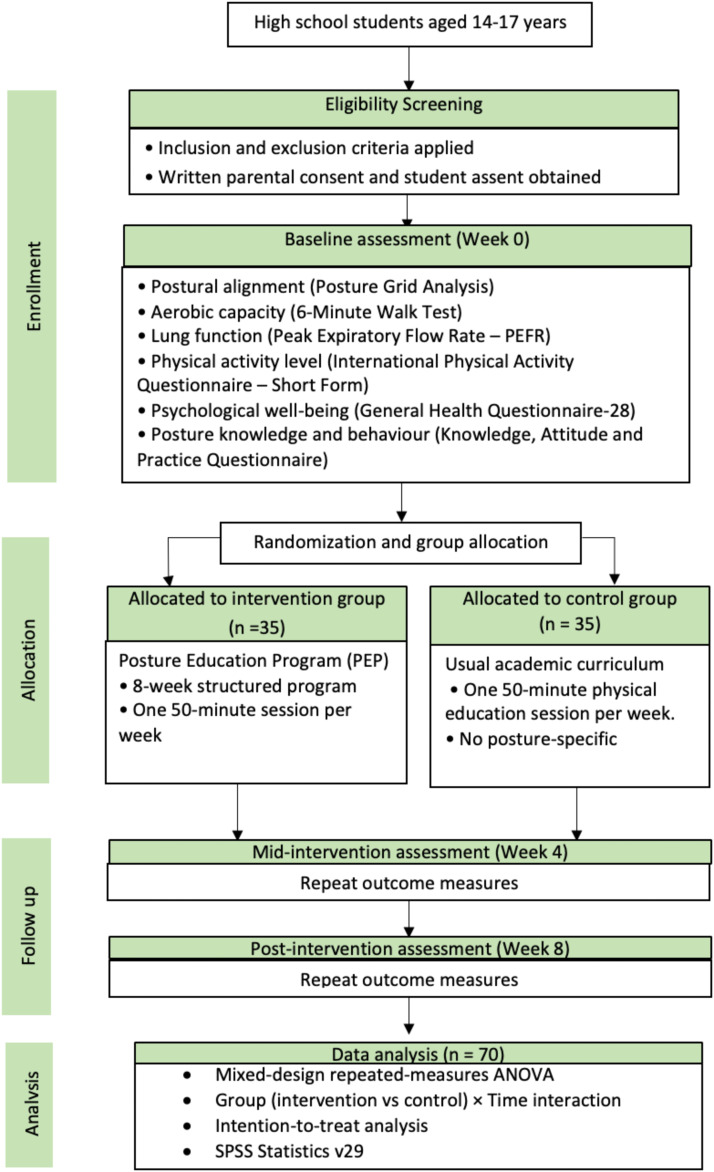
Flow chart of the study.

Similarly, previous school-based physical activity interventions among adolescents have demonstrated improvements in aerobic fitness and physical activity participation [7]. Nevertheless, many of these interventions primarily focused on general physical activity promotion and cardiovascular fitness rather than structured posture education, ergonomic correction, or posture-related behavioural modification. In addition, posture-related intervention studies frequently assessed musculoskeletal or alignment outcomes independently without integrating multidimensional physiological and psychological assessments Fig. 1.

Previous studies have also utilized posture assessment methods, respiratory outcome measures, and exercise capacity testing separately in adolescent populations [[8], [9], [10],^4^]. However, few published protocols have combined posture education, progressive postural exercise, ergonomic behaviour training, and multidimensional assessment of postural, cardiorespiratory, behavioural, and psychological outcomes within a single school-based randomized controlled trial. Furthermore, preventive posture education interventions targeting generally healthy adolescents within routine physical education settings remain limited and insufficiently described in the literature.

The present protocol addresses these gaps by providing a structured, theory-informed, and reproducible posture education intervention that can be integrated into scheduled physical education sessions without major disruption to the school timetable.

The novelty of the present protocol lies in its integration of posture education, ergonomic behaviour training, progressive postural exercise, and multidimensional outcome assessment within a structured school-based randomized controlled trial. The protocol combines objective assessment of posture, aerobic capacity, and lung function with behavioural and psychological outcome measures . In addition, the use of the ADDIE instructional framework supports systematic intervention development and implementation planning, while the ICF framework supports alignment of intervention components with body functions, activity, participation, and contextual domains [11]. This combined theory-informed approach provides a reproducible model for evaluating posture education as a preventive intervention among generally healthy high school students.

There is a need for a structured, reproducible protocol that combines posture education with multidimensional outcome assessment aligned with contemporary health models. The posture education program (PEP) was developed using the Analysis, Design, Development, Implementation, and Evaluation (ADDIE) instructional framework and validated through expert consensus using a Delphi process. The conceptual structure of the program is informed by the International Classification of Functioning, Disability, and Health (ICF) framework [11], ensuring alignment across domains of body functions, body structures, activity, and participation. The ADDIE framework was used to systematically guide the development and implementation of the intervention, while the ICF framework informed the classification of outcome domains, ensuring that intervention components target measurable changes in body functions, activity, and participation.

The purpose of this protocol article is to describe, in detail, the implementation procedures and standardized assessment methods for a randomized controlled trial evaluating the PEP in adolescents aged 14–17 years in the United Arab Emirates. The protocol provides sufficient methodological detail to enable replication in similar educational settings and supports comprehensive evaluation of posture, aerobic capacity, psychological well-being, and ergonomic knowledge.

## Method details

### Study design

This study employs a two-arm, parallel-group, randomized controlled trial (RCT) design with a 1:1 allocation ratio. The intervention group receives the PEP delivered during scheduled physical education sessions, replacing the standard PE content, while the control group continues with the standard PE curriculum alone.

The protocol was developed in accordance with the Standard Protocol Items: Recommendations for Interventional Trials (SPIRIT) guidelines for clinical trial protocols. Although the intervention is delivered in a school setting, participant-level randomization was selected because this study is designed as a preliminary randomized controlled protocol evaluating the feasibility and effectiveness of the PEP within scheduled physical education sessions. Both groups will receive sessions of equal duration and frequency to minimize attention-related bias. However, due to the educational and exercise-based nature of the intervention, complete blinding of participants and educators is not feasible.

### Study location

A secondary school in the United Arab Emirates.

### Sample size calculation

Sample size was calculated using G*Power version 3.1 for a mixed-design repeated-measures ANOVA examining the group × time interaction. Peak expiratory flow rate (PEFR) was selected as the primary outcome for sample size estimation. The selection of the effect size was informed by a previous randomized controlled trial investigating the effect of McKenzie exercises on pulmonary function among individuals with forward head posture, which demonstrated significant improvements in PEFR following the intervention [12]. To avoid overestimating the intervention effect and to ensure a conservative estimate for the present school-based posture education programme, a medium effect size (f = 0.25), alpha level of 0.05, and statistical power of 0.80 were used for the sample size calculation. The analysis indicated that a minimum of 28 participants per group was required. Allowing for an anticipated attrition rate of 20%, the target sample size was increased to 35 participants per group, resulting in a total planned sample of 70 students.

### Inclusion and exclusion criteria

Participants eligible for inclusion will be students aged 14 to 17 years who provide written parental or legal guardian consent and student assent prior to participation, and who are willing and able to attend the scheduled posture education program sessions and outcome assessment time points.

Students with diagnosed cardiovascular, respiratory, musculoskeletal, or neurological conditions that may limit safe participation in physical activity, such as severe scoliosis requiring medical treatment, uncontrolled asthma, recent musculoskeletal injury, or neurological disorders affecting mobility, will be excluded.

Eligibility screening will be conducted by the research team prior to baseline assessment.

### Randomization and blinding

Participants will be randomly assigned to either the intervention or control group using a computer-generated block randomization sequence with variable block sizes of four and six to ensure balanced allocation between groups throughout the recruitment period while minimizing predictability of group assignment. Randomization will be performed by an independent researcher not involved in data collection or intervention delivery. Allocation concealment will be ensured using sequentially numbered sealed opaque envelopes prepared before participant enrolment. Outcome assessors will be blinded to group allocation to minimize measurement bias. Participants will not be informed of the study hypotheses and will attend sessions as part of their scheduled school activities to minimize expectation bias. To ensure blinding, assessors will not be involved in intervention delivery and will be instructed not to ask participants about their group allocation. Participants will also be reminded not to disclose their group assignment during assessments. Any accidental unblinding during outcome assessment procedures will be documented.

### Intervention

Participants allocated to the intervention group will receive the 8-week PEP delivered during scheduled physical education sessions, consisting of one 50-minute session per week. To ensure feasibility within the school setting and minimize disruption to academic activities, sessions will be conducted during scheduled physical education periods. Each session will involve approximately 15 to 20 students and will be supervised by a trained educator to ensure correct exercise performance, safety, and adherence to the intervention protocol. Both groups will receive sessions of equal frequency and duration (one 50-minute session per week); however, only the intervention group will receive structured posture education content. The program includes postural awareness training, core strengthening exercises, ergonomic correction strategies, guided movement activities, and breathing and relaxation techniques. Participants’ baseline posture control, and core endurance will be assessed during the initial session to guide appropriate exercise starting levels. All participants will begin with low-intensity posture-focused activation exercises to ensure familiarization with correct movement techniques.

Progressive overload will be applied throughout the intervention by gradually increasing exercise hold duration, number of repetitions, sets, and movement complexity in accordance with established physical activity and exercise guidelines [13]. Functional postural control tasks and coordinated movement patterns will be introduced in the later blocks to enhance transfer of learning to daily activities. Exercise modifications will be provided based on individual tolerance and performance to ensure safe and standardized participation. Participant progression will be monitored weekly using standardized educator observation checklists to document exercise performance quality, tolerance, and adherence to the program. To facilitate gradual adaptation and skill acquisition, the PEP is delivered in four progressive blocks across the 8-week intervention period. The structure and progression of intervention components across these blocks are summarized in Table 1. Each PEP session will follow a standardized 50-minute structure consisting of postural awareness activities, core strengthening exercises, ergonomic correction practice, guided movement activities, and breathing and relaxation exercises. Exercises will include abdominal bracing, pelvic tilts, scapular setting, chin tuck exercises, wall alignment drills, bridge exercises, modified plank holds, bird-dog progression activities, thoracic mobility exercises, and functional posture-control tasks. Initial intensity will emphasize correct movement technique, controlled breathing, posture awareness, and low-intensity activation exercises. Progression across the intervention blocks will occur through gradual increases in exercise hold duration, repetitions, sets, movement complexity, coordination demands, and reduction in verbal cueing. Detailed exercise descriptions, session progression parameters, and FITT-based exercise implementation details are provided in Supplementary File 1 to enhance reproducibility and methodological transparency Table 2.

**Table 1:** Block-wise progression of the PEP.

Session Component	Duration (minutes)	Block 1 (Weeks 1–2)	Block 2 (Weeks 3–4)	Block 3 (Weeks 5–6)	Block 4 (Weeks 7–8)
Postural awareness training	10	Technique learning	Posture correction	Self-monitoring skills	Habit integration
Core strengthening exercises	15	Static activation	Endurance progression	Dynamic control	Functional stability
Ergonomic correction strategies	5	Ergonomic education	Practice and feedback	Behaviour application	Independent management
Guided movement activities	15	Mobility training	Alignment control	Coordination tasks	Functional movement
Breathing and relaxation techniques	5	Basic breathing	Posture-linked breathing	Movement integration	Self-regulation

Note: Total session duration = 50 minutes. The PEP is delivered once per week for 8 weeks during scheduled physical education sessions.

**Table 2:** Mapping of the ADDIE framework to the development and implementation of the PEP.

ADDIE phase	Application in the PEP
Analysis	Identification of adolescent postural risks, sedentary behaviour, ergonomic needs, and school-based feasibility requirements through literature review, school-context evaluation, and preliminary needs assessment to guide intervention planning.
Design	Development of intervention objectives, session structure, progression blocks, and outcome domains through expert-informed planning, evidence-based exercise selection, and Delphi content validation procedures.
Development	Preparation of educational materials, exercise components, observation checklists, and assessment procedures
Implementation	Delivery of one 50-minute PEP session weekly for 8 weeks during scheduled physical education sessions
Evaluation	Delphi content validation, pilot feasibility testing, fidelity monitoring, and outcome assessment at baseline, Week 4, and Week 8

### Description of intervention phases

#### Block 1 (Weeks 1–2): technique learning and familiarization

During the initial block, participants will focus on technique learning and familiarization with correct postural alignment. Activities include basic postural awareness exercises, static core activation (e.g., abdominal bracing and scapular setting), ergonomic education on sitting and standing posture, and simple mobility exercises. Breathing techniques emphasize diaphragmatic breathing to promote relaxation and postural control.

#### Block 2 (Weeks 3–4): posture correction and endurance development

In this block, participants will engage in posture correction exercises with increased focus on maintaining alignment during sustained positions. Core strengthening exercises progress to endurance-based tasks, with longer hold durations and increased repetitions. Ergonomic strategies are reinforced through practice and feedback, and guided movement activities target alignment control during functional movements. Breathing is integrated with posture correction tasks.

#### Block 3 (Weeks 5–6): dynamic control and coordination

This block introduces dynamic control and coordination tasks. Participants perform exercises requiring maintenance of postural alignment during movement, including coordinated movement patterns and functional tasks. Core exercises progress to dynamic control activities, and participants begin applying ergonomic principles in simulated daily activities. Movement–breathing integration is emphasized.

#### Block 4 (Weeks 7–8): functional integration and self-management

In the final block, participants focus on functional integration and independent self-management of posture. Exercises incorporate functional stability tasks and real-life movement patterns. Participants are encouraged to self-monitor posture and apply ergonomic strategies independently. Breathing and relaxation techniques support self-regulation and long-term adherence to correct postural behaviors.

Exercise progression across blocks is guided by participants’ baseline posture control and core endurance assessed during the initial session. Intensity and complexity increase gradually with longer hold durations, higher repetitions, greater coordination demands, and functional posture tasks. Weekly educator observation checklists are used to monitor performance quality, tolerance, and adherence, and exercise modifications are provided when necessary. Detailed examples of session structure, exercise progression, and instructional components are provided in Supplementary File 1.

Participants in the control group will continue their usual academic curriculum and attend the standard school physical education (PE) program, consisting of one 50-minute session per week. Trained PE teachers conduct the PE sessions and typically include general warm-up exercises, team sports activities such as football or basketball, basic fitness exercises including running and stretching, and recreational movement tasks. The sessions are designed to promote general physical fitness and participation rather than structured posture training or targeted core stabilization exercises. No specific posture education or ergonomic training components will be delivered to the control group during the study period.

### Outcome measures

Standardized outcome assessments will be conducted at baseline (Week 0), mid-intervention (Week 4), and post-intervention (Week 8). All assessments will be performed at approximately the same time of day to minimize physiological variability.

Primary outcome measures include lung function, postural alignment, and functional aerobic capacity. Postural alignment will be assessed using standardized posture grid analysis based on validated observational criteria [9,10]. Participants will stand in a standardized anatomical position in front of a calibrated posture grid, and alignment will be evaluated relative to vertical and horizontal reference lines to identify deviations in head position, shoulder symmetry, and spinal curvature. Posture grid assessment has demonstrated acceptable inter-rater and intra-rater reliability in previous adolescent posture assessment studies [9,10]. Standardized assessor training and assessment procedures will be implemented to support consistency during data collection.

Functional aerobic capacity will be assessed using the six-minute walk test (6MWT) conducted according to established guidelines [14]. Participants will be instructed to walk as far as possible along a standardized corridor for six minutes with consistent verbal encouragement. The total distance covered in meters will be recorded as the outcome measure. In addition to walking distance, participants’ heart rate, blood pressure, and oxygen saturation (SpO₂) will be measured before, during, and after the test. Perceived exertion will be assessed using Borg’s Rating of Perceived Exertion (RPE) scale, a validated tool used to measure subjective exercise intensity during physical activity [15]. The 6MWT has demonstrated good reliability and validity for assessing submaximal exercise capacity in adolescent populations [8,16]. Changes in walking distance will be interpreted alongside baseline performance and age-appropriate normative reference values derived from healthy children and adolescents [17].

Lung function will be evaluated using peak expiratory flow rate (PEFR) measured with a calibrated peak flow meter [18,4]. Participants will perform three maximal expiratory efforts from a standing position following standardized instructions, and the highest recorded value (L/min) will be used for analysis. PEFR is a widely accepted indicator of ventilatory capacity and pulmonary function in adolescent respiratory assessment. PEFR values will be interpreted with consideration of participant age, sex, and anthropometric characteristics where appropriate. Because PEFR is effort-dependent, standardized instructions, demonstration procedures, and repeated trials will be used to improve measurement consistency. PEFR values will be interpreted using age- and sex-appropriate normative reference values where available [18,4]. Although universally established MCID values for PEFR in healthy adolescent populations remain limited, changes in PEFR will be interpreted alongside baseline performance and repeated assessment trends. PEFR measurement has demonstrated acceptable validity and test–retest reliability in adolescent respiratory assessment when standardized instructions and repeated trials are applied [18,4].

Secondary outcome measures include resting cardiovascular parameters, physical activity level, psychological well-being, and ergonomic knowledge and behaviour. Resting blood pressure and heart rate will be measured using a validated automatic digital monitor following standardized procedures [5]. Physical activity level will be assessed using the International Physical Activity Questionnaire Short Form (IPAQ-SF), which evaluates time spent in walking, moderate-intensity activity, vigorous-intensity activity, and sedentary behaviour over the previous seven days [19]. It has been shown to be valid and reliable across multiple populations [19]. Psychological well-being will be assessed using the General Health Questionnaire-28 (GHQ-28), a self-report screening instrument comprising four domains, including somatic symptoms, anxiety and insomnia, social dysfunction, and depression [20]. The GHQ-28 has demonstrated good reliability and validity across different populations [20]. Knowledge, attitude, and practice related to ergonomic behaviour will be evaluated using a validated adolescent-adapted questionnaire assessing posture awareness, ergonomic practices, and behavioural habits in educational settings [21,22].

### Data analysis

Data will be analyzed using IBM SPSS Statistics version 29.0 (IBM Corp., Armonk, NY, USA). Baseline characteristics will be summarized using descriptive statistics, including mean and standard deviation for continuous variables and frequencies and percentages for categorical variables. The primary analysis will follow the intention-to-treat (ITT) principle, whereby all randomized participants will be analyzed according to their originally allocated groups regardless of intervention adherence or withdrawal where possible. The ITT approach was selected to preserve the benefits of randomization, minimize attrition-related bias, and provide a pragmatic estimate of intervention effectiveness within a real-world school-based setting.

Missing data patterns will be examined, and the extent and reasons for missing data will be reported. Missing outcome data will be handled using appropriate imputation procedures, where necessary, to facilitate the inclusion of all randomized participants in the primary analysis, consistent with recommended approaches for randomized controlled trial analysis [23].

A mixed-design repeated-measures ANOVA will be used to examine group (intervention vs control) × time (baseline, mid-intervention [Week 4], and post-intervention [Week 8]) interaction effects for primary and secondary outcomes. Assumptions of normality, homogeneity of variance, and sphericity will be assessed using appropriate diagnostic procedures. Where the assumption of sphericity is violated, Greenhouse–Geisser correction will be applied. Significant interaction effects will be explored using post hoc pairwise comparisons with adjustment for multiple comparisons. Effect sizes for repeated-measures ANOVA will be reported using partial eta squared (η²p), while Cohen’s d will be calculated for post hoc pairwise comparisons, consistent with commonly recommended effect size reporting practices for intervention studies [12]. Statistical significance will be set at p < 0.05.

### Safety and adverse events

The PEP consists of low-risk educational and exercise-based activities appropriate for adolescents. However, potential minor adverse events such as temporary muscle soreness, fatigue, or mild discomfort during exercise may occur.

To minimize risks, all activities will be conducted under the supervision of trained educators, and participants will receive clear instructions on proper exercise techniques. Participants will be advised to stop any activity if discomfort or pain occurs. Any adverse events reported during the intervention period will be documented and monitored. If necessary, participants will be referred to appropriate school health personnel or medical services.

## Data confidentiality

All participant information will be treated as confidential. Each participant will be assigned a unique identification code, and personal identifying information will not be included in the dataset used for analysis.

Data will be stored in password-protected electronic files accessible only to the research team. Hard-copy documents, if any, will be stored securely in locked cabinets. The collected data will be used solely for research purposes and will not be shared with unauthorized individuals.

## Method validation

### Content validity

The Posture Education Program (PEP) was developed using the ADDIE instructional framework and underwent content validation through a Delphi consensus process involving 11 experts, including 2 physiotherapists, 5 educators, 2 physical education teachers, and 2 school nurses. The experts evaluated the relevance, clarity, comprehensiveness, safety, feasibility, and progression structure of the intervention components. Consensus was defined a priori as ≥80% agreement for each intervention component. The Delphi process was conducted across two iterative rounds. Feedback obtained during the first-round informed refinements to session content, ergonomic education materials, exercise progression strategies, instructional cues, safety considerations, and delivery procedures. The second Delphi round confirmed the acceptability and content relevance of the revised intervention program for implementation within a school-based adolescent population.

### Feasibility and fidelity

A pilot feasibility study was conducted prior to the main randomized controlled trial to assess the practicality, safety, acceptability, and fidelity of the PEP within a secondary school setting. A small group of eligible high school students participated in the pilot implementation over the planned intervention period. Feasibility indicators included participant attendance, session completion, participant engagement, educator adherence to the intervention protocol, completion of outcome assessments, and adverse event monitoring. The pilot feasibility phase was conducted to evaluate implementation readiness and refine procedural aspects of the PEP prior to progression to the full randomized controlled trial.

The pilot demonstrated good overall feasibility, with satisfactory participant attendance, consistent educator adherence to intervention procedures, successful completion of planned outcome assessments, and no reported adverse events. Educator observation checklists confirmed consistent delivery of intervention components and appropriate participant progression across sessions. The pilot also confirmed that the 50-minute session duration was practical within the school timetable and that assessment procedures could be completed efficiently within the school environment. Minor refinements were subsequently made to session sequencing, instructional cues, and assessment logistics prior to implementation of the main trial.

### Preliminary implementation observations

Baseline and post-intervention assessments were completed during the pilot phase to evaluate the practicality of data collection procedures, including posture grid analysis, peak expiratory flow rate measurement, six-minute walk testing, and questionnaire administration. The pilot confirmed that all outcome measures were feasible to administer within the school setting and acceptable to participants. Minor refinements were made to session sequencing, instructional cues, and assessment logistics prior to implementation of the main trial.

### Stakeholder acceptability

Informal feedback from participating students and teachers indicated that the PEP was well accepted and considered relevant to students’ daily postural habits, school ergonomics, and overall health awareness. Feedback supported the clarity of educational content, practicality of exercises, and appropriateness of session duration within the school timetable.

## Limitations

This study has several limitations. First, the intervention duration is limited to eight weeks, which may restrict evaluation of long-term behavioural changes and sustained physiological effects. Second, some secondary outcomes rely on self-reported measures, including the IPAQ-SF and GHQ-28, which may be influenced by recall bias, social desirability bias, and variability in adolescents’ interpretation of questionnaire items [19,20]. Third, because the intervention is delivered within a school setting, contamination between intervention and control participants may occur through peer interaction outside intervention sessions. Fourth, complete blinding of participants and educators was not feasible due to the educational and exercise-based nature of the intervention. Fifth, although participant-level randomization is used, the group-based school delivery format may introduce teacher-level or class-level influences that are not fully controlled. Finally, findings may have limited generalizability beyond generally healthy adolescents aged 14–17 years within the specific cultural and educational context of the United Arab Emirates. Future studies may consider cluster-randomized designs to better minimize potential contamination and class-level influences within school-based interventions, as well as longer follow-up periods and implementation across multiple schools and educational settings.

## Ethics statements

This study received ethical approval from the Universiti Kebangsaan Malaysia Research Ethics Committee (UKM REC; Ref No. JEP-2025-284). Ethical approval was granted on 10 July 2025 and is valid until 9 July 2028. Written informed consent will be obtained from parents or legal guardians, and assent will be obtained from participating students prior to enrolment. Participants may withdraw at any time without penalty.

## CRediT author statement

**Jeyanthi Valaitham:** Conceptualization, Methodology, Investigation, Writing – original draft, Writing – review & editing, Project administration, Formal analysis, Visualization, Resources, Data curation, Validation. **Asfarina Zanudin:** Conceptualization, Methodology, Supervision, Writing – review & editing, Validation. **Nurfaradilla Mohamad Nasri:** Supervision, Writing – review & editing, Validation. **Ayiesah Ramli:** Supervision, Writing – review & editing, Validation.

## Declaration of competing interest

The authors declare that they have no known competing financial interests or personal relationships that could have appeared to influence the work reported in this paper.

## Data Availability

Data will be made available on request.

## Appendix Supplementary materials

Image, application 1
