# Supplementary material for: Effectiveness of a posture education program in high school students: A randomized controlled trial protocol
Source: MethodsX. 2026 Jun 13;17:104006. doi: 10.1016/j.mex.2026.104006 (PMC13314789; doi:10.1016/j.mex.2026.104006)
Supplement: Supplementary file 1 [file mmc1.docx]

**Supplementary File 1. Sample Session Structure and Exercise Examples for the Posture Education Program (PEP)**

This supplementary file provides additional examples of session structure, exercise descriptions, progression strategies, and fidelity monitoring procedures used within the Posture Education Program (PEP).

Overview

The Posture Education Program (PEP) is delivered during scheduled physical education sessions and consists of one 50-minute session per week over an 8-week period. The intervention combines postural awareness training, ergonomic education, core strengthening, guided movement activities, and breathing and relaxation exercises. Progression is implemented gradually across four intervention blocks to ensure safe adaptation, skill acquisition, and functional integration.

Supplementary Table 1. Sample 50-Minute PEP Session Structure

| **Session Component** | **Duration** | **Example Activities** | **Purpose** |
| --- | --- | --- | --- |
| Introduction and posture goal setting | 5 min | Brief discussion on posture awareness and daily ergonomic habits | Improve engagement and session focus |
| Postural awareness activities | 10 min | Mirror posture correction, wall alignment drills, seated posture practice | Enhance awareness of neutral alignment |
| Core strengthening exercises | 15 min | Abdominal bracing, bridges, bird-dog exercises, modified planks | Improve trunk stability and postural support |
| Ergonomic correction practice | 5 min | Backpack positioning, desk/chair posture correction, device positioning | Promote ergonomic behaviour |
| Guided movement activities | 10 min | Sit-to-stand control, balance tasks, coordinated movement patterns | Improve movement control and posture integration |
| Breathing and relaxation exercises | 5 min | Diaphragmatic breathing, posture-linked breathing, relaxation exercises | Support breathing control and self-regulation |

**Example Exercise Descriptions**

Supplementary Table 2. FITT-Based Exercise Examples and Progression Parameters for the PEP

| **Exercise** | **Purpose** | **Procedure** | **Frequency** | **Intensity** | **Duration / Repetitions** | **Progression** |
| --- | --- | --- | --- | --- | --- | --- |
| Abdominal bracing | Improve core activation and spinal stability | Participants gently contract abdominal muscles while maintaining neutral spine posture and normal breathing | Once weekly within supervised PEP sessions | Low intensity | 2–3 sets, 5 repetitions, 5–15 second holds | Hold duration gradually increased across blocks |
| Chin tuck | Improve cervical posture awareness | Participants retract the head backward while maintaining neutral gaze | Once weekly | Low intensity | 8–10 repetitions | Combined with wall alignment drills |
| Scapular setting | Improve shoulder alignment and scapular control | Participants retract and depress scapulae in upright posture | Once weekly | Low-to-moderate intensity | 5–10 second holds, 8 repetitions | Progressed to movement integration tasks |
| Bridge exercise | Improve gluteal and trunk activation | Participants lift pelvis from crook-lying position maintaining neutral alignment | Once weekly | Moderate intensity | 2 sets, 8–10 repetitions | Progressed to longer holds or single-leg variations |

Supplementary Table 3. Progression Across Intervention Blocks

| **Intervention Block** | **Progression Focus** |
| --- | --- |
| Weeks 1–2 | Familiarization, posture awareness, low-intensity activation |
| Weeks 3–4 | Increased endurance, posture correction, ergonomic application |
| Weeks 5–6 | Dynamic control, coordinated movement, functional posture tasks |
| Weeks 7–8 | Functional integration, self-monitoring, independent posture management |

**Fidelity and Monitoring Procedures**

Standardized educator observation checklists are used during each session to monitor intervention fidelity, participant safety, and progression throughout the program.

Supplementary Table 4. Educator Observation Checklist for Fidelity and Monitoring Procedures

| **Monitoring Component** | **Detailed Monitoring Procedure** |
| --- | --- |
| Exercise performance quality | Educators observe participants’ posture alignment, movement control, breathing coordination, exercise technique, and ability to maintain correct positioning during exercises. Incorrect movements or compensatory patterns are identified, and immediate verbal feedback and demonstration are provided to improve exercise performance consistency. |
| Participant tolerance and safety | Educators continuously monitor participants for signs of discomfort, pain, excessive fatigue, dizziness, shortness of breath, or exercise intolerance during sessions. Participants are encouraged to report discomfort immediately, and exercises are modified, paused, or discontinued when necessary to maintain safety. |
| Session adherence | Attendance and completion of planned activities are recorded during each supervised session. Educators document participant participation, incomplete activities, missed sessions, and reasons for non-compliance where applicable. |
| Progression readiness | Before progressing exercises, educators assess whether participants can perform activities with appropriate posture control, movement quality, coordination, and tolerance. Progression includes gradual increases in exercise hold duration, repetitions, coordination demands, or movement complexity according to individual performance. |
| Participant engagement | Educators observe participant attention, cooperation, responsiveness to instructions, willingness to participate, and overall involvement during session activities to support consistent engagement and learning throughout the intervention. |

Exercise modifications are provided when necessary to ensure safe participation and appropriate progression according to individual tolerance and performance level.
